# Effects of land use change from natural forest to plantation on C, N and natural abundance of ^13^C and ^15^N along a climate gradient in eastern China

**DOI:** 10.1038/s41598-019-52959-z

**Published:** 2019-11-11

**Authors:** Mbezele Junior Yannick Ngaba, Ya-Lin Hu, Roland Bol, Xiang-Qing Ma, Shao-Fei Jin, Abubakari Said Mgelwa

**Affiliations:** 1 Forest Ecology and Stable Isotope Research Center, College of Forestry, Fujian Agriculture and Forestry University, Fuzhou, 350002 People’s Republic of China; 2 Agrosphere (IBG-3), Institute of Bio- and Geosciences, Forschungszentrum Jülich GmbH, 52428 Juelich, Germany; 3 Department of geography, Minjiang University, Fuzhou, 350108 People’s Republic of China; 4 College of Natural Resources Management & Tourism, Mwalimu Julius K. Nyerere University of Agriculture & Technology, P.O. Box 976 Musoma, Tanzania

**Keywords:** Forest ecology, Forestry, Stable isotope analysis

## Abstract

Soil C and N turnover rates and contents are strongly influenced by climates (e.g., mean annual temperature MAT, and mean annual precipitation MAP) as well as human activities. However, the effects of converting natural forests to intensively human-managed plantations on soil carbon (C), nitrogen (N) dynamics across various climatic zones are not well known. In this study, we evaluated C, N pool and natural abundances of *δ*^13^C and *δ*^15^N in forest floor layer and 1-meter depth mineral soils under natural forests (NF) and plantation forest (PF) at six sites in eastern China. Our results showed that forest floor had higher C contents and lower N contents in PF compared to NF, resulting in high forest floor C/N ratios and a decrease in the quality of organic materials in forest floor under plantations. In general, soil C, N contents and their isotope changed significantly in the forest floor and mineral soil after land use change (LUC). Soil *δ*^13^C was significantly enriched in forest floor after LUC while both *δ*^13^C and *δ*^15^N values were enriched in mineral soils. Linear and non-linear regressions were observed for MAP and MAT in soil C/N ratios and soil *δ*^13^C, in their changes with NF conversion to PF while soil *δ*^15^N values were positively correlated with MAT. Our findings implied that LUC alters soil C turnover and contents and MAP drive soil *δ*^13^C dynamic.

## Introduction

Globally, soil is the largest carbon (C) pool in terrestrial ecosystems^1^. Approximately 1,500 ± 230 Gt of soil organic carbon (SOC) is stored in the first meter of soil, nearly twice as much as atmospheric carbon (828 Gt as CO_2_)^2^ and almost 44% is stored in forests^3^. A better understanding of soil C and N balance in forest floor and mineral soil can help guide the implementation of mitigation policies to reduce the emission of greenhouse gases by sources or enhance their removal from the atmosphere by “sinks” which refers to forests, vegetation or soils that can reabsorb CO_2_^3,4^. In contrast to the clear inventory-based assessments of aboveground C on both regional^5^ and global scales^3^, the C and N pools in forest floor and mineral soils remain uncertain because of the higher variations among global forest ecosystems^6,7^.

Natural environmental factors and human activities generate abrupt, large scale, irreversible changes and thus alters forest composition^8,9^, consequently resulting in the changes of soil C and N cycles^10–12^. The impacts of converting natural forest (NF) to plantation forest (PF) on soil C and N cycles have been well examined at the stand scales, relating their variances in above- and below-ground C input through litter-fall, root exudation, and their inherently different management practices^13–15^. However, the influence of the wider regional or global scale conversion of NF to PF on changes of soil C and N cycles is not yet well understood. This is perceived as a key bottleneck in improving the prediction and feedback of soil C and N mitigation related to land use change (LUC). Previous studies have suggested that the impact of converting natural forests to plantations significantly impacts the quantity and quality of C and N input between natural forests and plantations^12,14,15^. Finzi *et al*.^10^ for example, reported that changes in the species composition control forest floor and surface soil C and N dynamics while converting natural evergreen broadleaf forests to moso bamboo plantations significantly decreased the concentrations of C within the 0–40-cm.

The natural abundance of soil ^13^C and ^15^N (expressed as °/_oo_) has uniquely been used to estimate soil C turnover rate^16–20^. The ^13^C and ^15^N abundance in soils is a dynamic function of the rate and isotopic composition of inputs and outputs, and the internal C, N transformations that occur in a soil system. It can also be used as a gross indicator of environmental processes that impact soil C storage in forest ecosystems and therefore provide integrated insights into soil C and N cycles^21,22^. Litter inputs generally lowers soil *δ*^13^C and *δ*^15^N values while higher decomposition leads to increased *δ*^13^C and *δ*^15^N values^21,23^. Moreover, it has been well evaluated that for sites with stable vegetation and low human disturbance, SOC turnover rates do not significantly change according to vertical soil-profile SOC contents and *δ*^13^C^20,22,24^. The values of soil *δ*^15^N vary with some soil N cycle processes, e.g., N deposition, soil N nitrification and denitrification, and are used as a tool to examine soil N availability^19,25,26^. In addition, N cycle processes in stand-scale forests altered by human disturbance and climatic factors^27,28^. However, details pertaining changing soil *δ*^13^C and *δ*^15^N with NF converted to intensively managed plantations along climatic gradients of MAT and MAP at a regional scale is still not well understood.

China, with a land area of 9.63 million km^2^ encompasses a climatic gradient, from cold temperate to tropical climate zones, and therefore has diverse land uses (LU) ranging from deciduous forests in the north to evergreen forests in the south^7,29^. China can be viewed as a unique “laboratory”, with its complex interactions between the varied climatic zones and intensive human activities, thus providing an excellent opportunity to examine simultaneous climate and human impacts on the forest soil C and N pools^30^. To date, several studies have been carried out to evaluate soil C pools across China’s forests using statistical or biogeochemical models^7,31,32^. However, there are some inconsistencies in soil C and N pool estimations such as the increasing of plant cover through reforestation and afforestation programs, the lack of data from repeated inventories, the variety of methods used to assess the carbon balance of China and the variation of climate conditions within those studies which vary from one period to another. Among others, these are either because of the insufficient observations or inconsistent measurement methods among the studies^32^. Recently, Tang *et al*.^33^ conducted a field survey involving 14,371 field plots to evaluate the current C stocks in mineral soil of China’s terrestrial ecosystems in order to examine their biogeographical patterns and potential climatic drivers. However, the aforementioned studies evaluated soil C pools only in the NF. The increasing needs for timber and other economic forest products in China, means that large areas of NF has been converted to intensively managed plantations^12,34,35^ but there is still significant uncertainty concerning changes in forest floor and mineral soil C and N stocks and turnover processes potentially induced by forest conversion along the 4200 km transect from northern China to southern China.

The objectives of this study are, in a range of climate zones in eastern China to: (i) determine the patterns of soil C and N contents (g kg^−1^) and *δ*^13^C and *δ*^15^N values for 1 meter vertical depth in NF and PF in forest floor and mineral soil, (ii) quantify changes in soil C and N contents and turnover rates as induced by LUC, and (iii) determine the effect of climate factors on the patterns of soil C and N contents, and natural abundance of soil ^13^C and ^15^N after LUC. We hypothesize that: (i) C, N, *δ*^13^C and *δ*^15^N contents in forest floor would significantly differ between NF and PF, and vary significantly among the study sites in the climate gradient, (ii) site specific content and turnover times of soil C and N would be increased by LUC, but (iii) overall climate controlled variations in soil C, N, *δ*^13^C and *δ*^15^N content between site would not be significantly affected.

## Results

### Carbon and nitrogen contents, and C/N ratios in forest floor and mineral soils

Forest floor C, N contents and C/N ratio were significantly different among the study sites (*P* < 0.001, for all except for N content *P* < 0.01) and their values were considerably affected following conversion of natural forest (NF) to plantation forest (PF) (Table 1). Furthermore, forest floor C mean content was lower in NF with 399.8 ± 86.1 g kg^−1^ across six sites as compared to PF with 438.6 ± 70.9 g kg^−1^ while an opposite trend was observed in forest floor N content with 13.6 ± 1.8 g kg^−1^ in NF and 10.3 ± 2.6 g kg^−1^ in PF. We observed an increase of forest floor C varying from 2–17% among sites following the order: DH > JF > MH > QY > HT > XY. Moreover, a general increase of C/N ratio and decrease of soil N in forest floor were observed except at XY site (Table 2).

**Table 1:** The two-way ANOVA results for all soil variables in forest floor.

	Variables	*F*	*P*
Sites	C	31.136	***
	N	5.363	**
	C/N	33.872	***
	^13^C	19.291	***
	^15^N	25.170	***
Land use	C	11.644	**
	N	34.502	***
	C/N	109.409	***
	^13^C	48.655	***
	^15^N	2.159	0.155
Sites × Land use	C	0.448	0.810
	N	5.234	**
	C/N	15.856	***
	^13^C	6.159	***
	^15^N	3.523	*

n = 6 (Sites), n = 18 (Land use);

*, ** and *** indicate a significant level at *P* < 0.05, *P* < 0.01 and *P* < 0.001, respectively.

**Table 2:** Mean values of C and N content (g kg^−1^) and C/N ratios in forest floor and mineral soil layers in natural forests and plantations at six sites across the eastern China.

			MH		QY		XY		HT		DH		JF	
			NF	PF	NF	PF	NF	PF	NF	PF	NF	PF	NF	PF
C	FF	Mean	417.8	448.1	378.6	421.1	239.5	305.4	430.1	479.8	474.2	485.4	458.5	491.5
		SD	30.8	3.4	34.5	16.6	71.6	68.9	9.8	18.1	25.6	10.3	19.4	7.9
	0–10 cm	Mean	49.55	41.51	48.95	41.06	18.61	21.29	17.09	15.15	25.77	14.67	25.38	19.39
		SD	15.23	10.54	30.83	21.11	4.84	4.77	4.14	3.20	1.93	4.34	2.54	0.24
	10–20 cm	Mean	30.19	12.64	26.67	23.41	7.98	12.24	10.03	12.87	12.53	5.05	15.83	12.48
		SD	15.76	5.63	6.19	1.70	4.35	4.96	0.53	2.49	2.55	0.58	5.54	0.75
	20–40 cm	Mean	11.86	3.50	12.84	12.99	3.50	6.62	8.54	9.43	6.97	3.93	11.00	6.93
		SD	2.84	0.58	2.59	2.22	1.61	2.34	1.62	1.45	0.61	0.48	3.99	1.16
	40–60 cm	Mean	4.27	3.81	6.97	5.82	2.57	4.69	5.47	6.02	5.14	3.03	6.58	6.21
		SD	1.44	0.67	0.67	0.54	1.07	0.47	1.62	2.53	0.59	0.44	0.95	1.29
	60–80 cm	Mean	3.30	3.86	4.38	3.86	1.88	3.50	4.54	5.07	4.70	6.21	4.61	8.52
		SD	0.49	0.41	0.96	0.66	0.91	1.35	1.45	3.06	0.82	2.78	0.12	5.20
	80–100 cm	Mean	4.51	3.92	2.76	3.07	2.21	2.98	4.06	4.31	3.57	3.41	3.34	3.28
		SD	2.08	0.47	1.24	0.80	1.13	0.79	1.36	1.51	0.36	0.85	0.92	0.65
N	FF	Mean	11.2	11.1	15.1	12.1	11.6	12.2	14.3	7.3	15.7	12.3	13.3	6.5
		SD	0.3	1.7	0.4	0.8	3.5	2.3	1.1	0.1	0.2	1.9	2.5	0.8
	0–10 cm	Mean	2.34	1.94	4.23	3.23	1.44	1.46	1.99	1.66	2.13	1.24	1.98	1.51
		SD	0.72	0.52	2.10	1.18	0.40	0.35	0.32	0.27	0.03	0.31	0.12	0.07
	10–20 cm	Mean	1.41	0.75	2.60	2.42	0.65	0.77	1.30	1.48	1.13	0.60	1.32	1.13
		SD	0.26	0.21	0.60	0.15	0.27	0.08	0.04	0.31	0.18	0.04	0.29	0.07
	20–40 cm	Mean	0.76	0.36	1.33	1.49	0.34	0.57	1.18	1.17	0.75	0.49	0.97	0.64
		SD	0.09	0.04	0.37	0.20	0.16	0.08	0.22	0.22	0.08	0.07	0.23	0.10
	40–60 cm	Mean	0.39	0.45	0.79	0.69	0.28	0.49	1.00	0.97	0.67	0.45	0.66	0.64
		SD	0.09	0.02	0.11	0.08	0.12	0.06	0.08	0.18	0.06	0.05	0.06	0.11
	60–80 cm	Mean	0.33	1.41	0.51	0.49	0.23	0.36	0.94	0.91	0.63	1.39	0.50	1.17
		SD	0.06	0.81	0.13	0.08	0.08	0.13	0.14	0.22	0.06	0.68	0.02	0.82
	80–100 cm	Mean	0.40	0.48	0.34	0.36	0.25	0.33	0.92	0.90	0.57	0.56	0.36	0.35
		SD	0.13	0.03	0.16	0.09	0.09	0.05	0.08	0.15	0.06	0.04	0.09	0.07
C:N	FF	Mean	21.5	21.5	25.1	35.1	20.6	24.9	30.2	65.3	30.1	40.1	35.1	75.7
		SD	5.2	1.7	3.1	2.2	0.2	1.3	2.4	3.4	2.1	6.9	6.1	11.1
	0–10 cm	Mean	21.00	16.20	11.07	12.33	13.11	14.61	8.50	9.15	12.09	11.72	12.82	12.82
		SD	7.97	2.87	1.51	1.74	1.60	1.38	0.93	1.52	1.00	0.87	1.04	0.37
	10–20 cm	Mean	16.00	9.71	10.27	9.69	12.00	15.63	7.71	8.74	11.02	8.40	11.82	11.10
		SD	5.15	0.67	0.69	0.13	5.31	4.88	0.23	0.91	0.57	0.46	1.49	0.58
	20–40 cm	Mean	10.74	8.48	9.78	8.70	10.36	11.38	7.25	8.10	9.28	8.09	11.12	10.86
		SD	1.95	1.47	1.05	0.42	0.82	2.87	0.80	0.52	0.74	0.46	1.94	0.30
	40–60 cm	Mean	10.04	4.07	8.94	8.41	9.27	9.64	5.46	6.01	7.72	6.77	10.00	9.74
		SD	0.33	3.58	0.78	0.93	1.40	0.21	1.42	1.53	0.16	1.02	0.59	0.67
	60–80 cm	Mean	10.98	8.12	8.69	7.96	7.69	9.60	4.77	5.26	7.41	4.58	9.18	8.50
		SD	2.66	0.95	0.48	0.31	1.92	0.31	0.99	1.98	0.67	0.53	0.39	2.83
	80–100 cm	Mean	37.09	41.29	8.20	8.45	8.45	8.92	4.38	4.70	6.36	6.12	9.24	9.41
		SD	3.22	6.36	0.39	0.65	1.65	1.18	1.21	0.84	1.22	1.53	0.43	0.26

Data is the mean value (n = 3); FF (forest floor), MH (Mohe), QY (Qingyuan), XY (Xinyang), HT (Huitong), DH (Dinghushan), JF (Jianfengling), NF (natural forests), PF (plantation forests).

**Figure 1:**
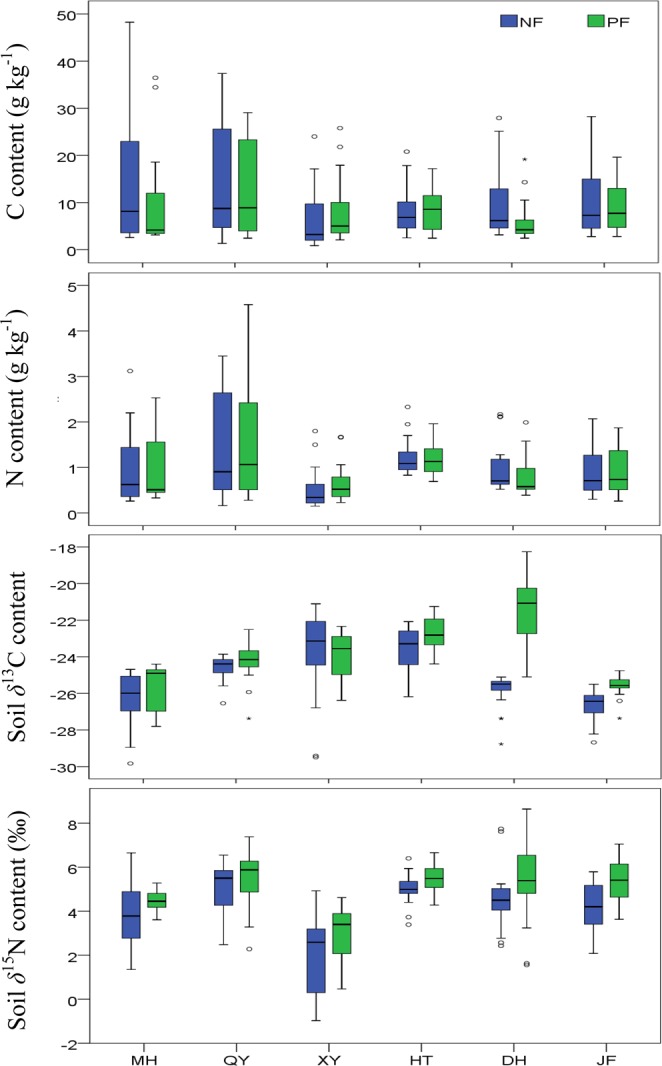
Median of (**a**) soil C content (g kg^−1^), (**b**) soil N content (g kg^−1^), (**c**) soil *δ*^13^C (‰) and (**d**) soil *δ*^15^N (‰) at 0–100 cm soil depth across different forest type and among sites. NF: Natural Forest; PF: Plantation Forest. A segment inside the rectangle shows the median. The boundary of the box indicates the distribution of soil samples (25th and 75th percentile). Error bars denote the maximum and minimum (90th and 10th percentiles).

Soil C ranged from 2.98 g kg^−1^ to 49.55 g kg^−1^ whereas soil N was in the range of 0.23 g kg^−1^ to 4.23 g kg^−1^ in mineral soil across the sampling sites (Table 2). There was a significant difference (*P* < 0.01) in soil C, N content and C/N ratio among sites (Table 3). The statistical analysis showed that soil C and C/N ratios were significantly (*P* < 0.05) altered following the conversion from NF to PF but soil N did not change significantly (*P* = 0.214). In addition, we observed a gain of the mean values of soil C content over 1 m by +6% (HT), +39% (XY) and a loss varied between −38% to −12% at DH > MH > JF > QY (Fig. 1a). Soil C/N ratio increased at XY, HT and JF sites (Table 2). On the other hand, although an increase of soil N contents after NF conversion to PF was observed at XY, there were no obvious differences among sites (Fig. 1b). Our results also indicated a decrease in soil C, N values and C/N ratio with soil depth. The statistical analyses affirmed this trend and revealed that soil depth significantly (*P < *0.01) alters C, N values and C/N ratio. However, the conversion from NF to PF strongly influenced C and C/N ratio (Table 3). In addition, a significant and positive correlation was found between C and N (*r* = 0.879*, P* < 0.01) (Table 4).

**Table 3:** The two-way ANOVA results for all soil variables over 1 m depth layer.

	Site		Depth		Land use		Site × Depth		Depth × Land use		Site × Land use	
	*F*	*P*	*F*	*P*	*F*	*P*	*F*	*P*	*F*	*P*	*F*	*P*
C	14.424	***	98.920	***	4.744	*	5.613	***	1.815	0.113	2.311	**
N	25.627	***	89.000	***	1.556	0.214	6.653	***	5.928	***	0.880	0.496
C/N	46.763	***	55.143	***	4.555	*	4.432	***	0.976	0.435	7.431	***
^13^C	104.483	***	69.494	***	144.399	***	4.419	***	4.205	***	45.629	***
^15^N	56.944	***	34.686	***	42.636	***	3.425	***	0.603	0.698	1.140	0.342

n = 36 (Site, depths), n = 108 (Land use);

*, ** and *** indicate a significant level at *p* < 0.05, *p* < 0.01 and *p* < 0.001, respectively.

**Table 4:** Pearson’s coefficients correlation between soil variables, land use and depth.

	C	N	C/N	^13^C	^15^N
C	/	0.879^**^	0.641^**^	−0.491^**^	−0.207^**^
N	0.879^**^	/	0.262^**^	−0.308^**^	−0.045
C/N	0.641^**^	0.262^**^	/	−0.606^**^	−0.377^**^
^13^C	−0.491^**^	−0.308^**^	−0.606^**^	/	0.369^**^
^15^N	−0.207^**^	−0,045	−0.377^**^	0.369^**^	/
LU	−0.073	−0.041	−0.074	0.304^**^	0.236^**^
Depth	−0.663^**^	−0.610^**^	−0.553^**^	0.355^**^	0.226^**^

^*^Correlation is significant at the 0.05 level (2-tailed).

**Correlation is significant at the 0.01 level (2-tailed).

n.s (not significant), n = 216, n = 108 (age), LU (land use), MAP (mean annual precipitation), MAT (mean annual temperature).

### The natural abundance of ^13^C and ^15^N in forest floor and mineral soils

**Figure 2:**
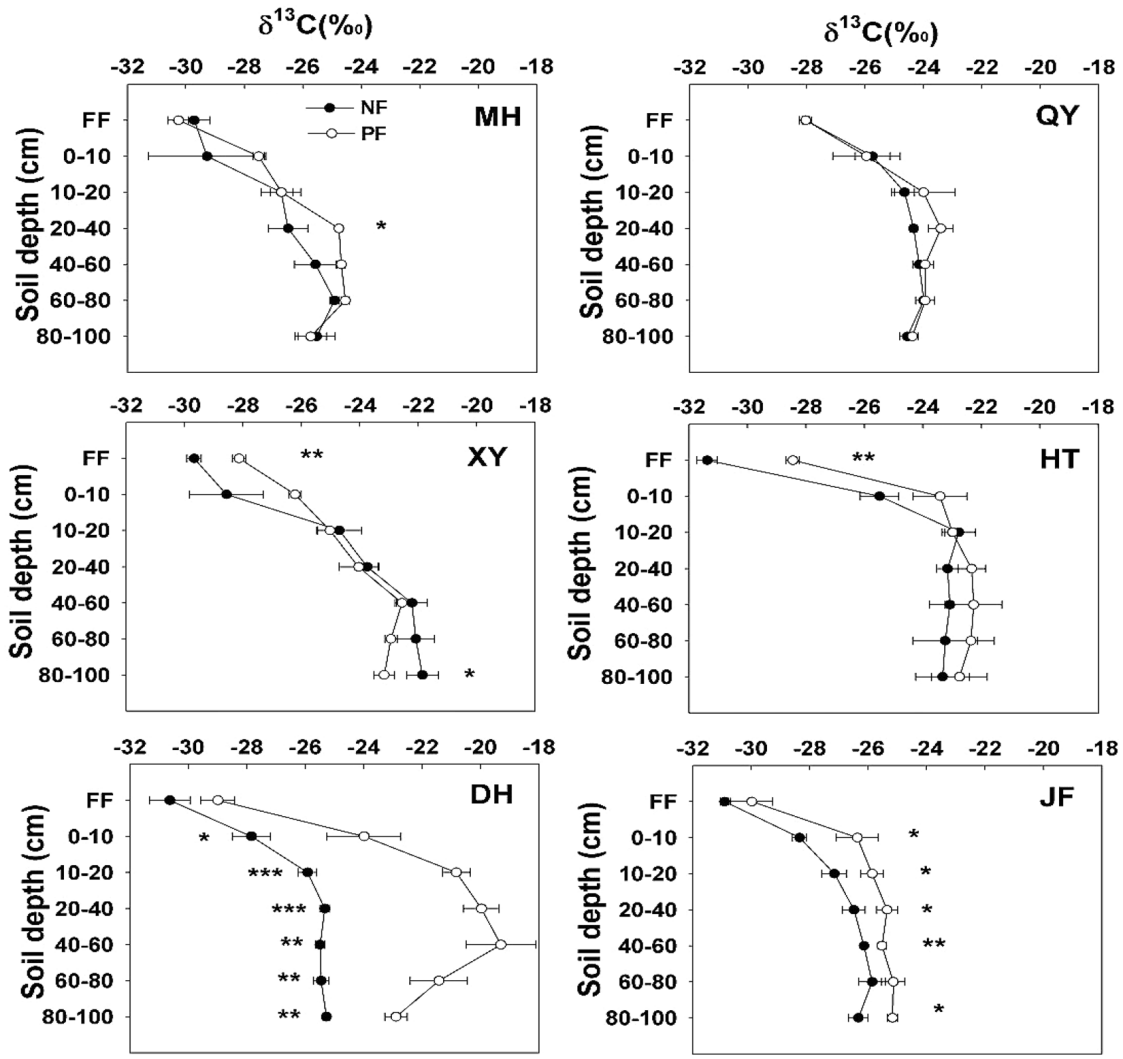
The values of *δ*^13^C in forest floor and mineral soil layers in natural forests and plantations at six sites across the eastern China. The error bars indicate standard deviation (n = 3). MH (Mohe), QY (Qingyuan), XY (Xinyang), HT (Huitong), DH (Dinghushan), JF (Jianfengling). *, ** and *** indicate a significant level at *p* < 0.05, *p* < 0.01 and *p* < 0.001, respectively.

**Figure 3:**
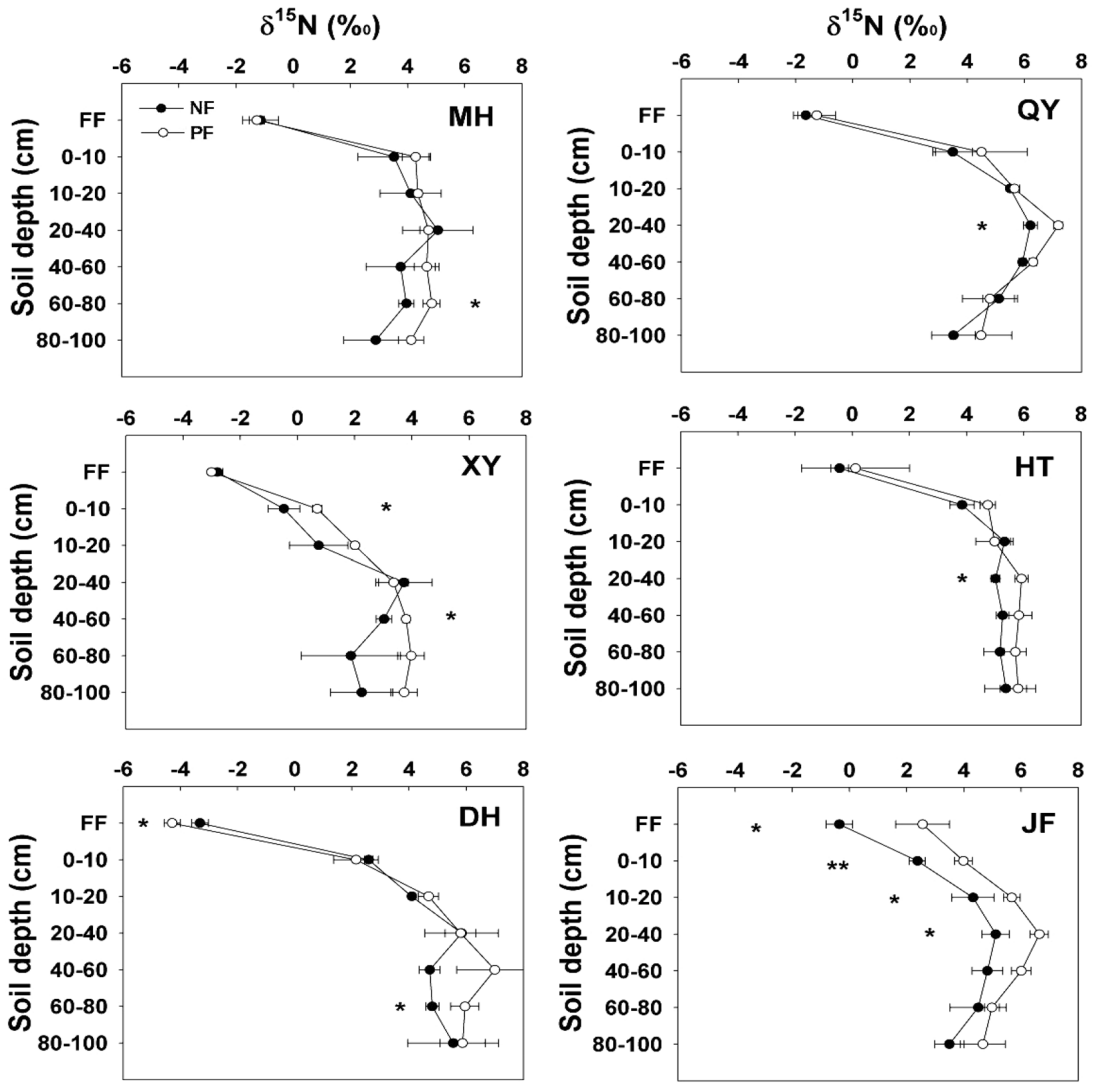
The values of *δ*^15^N in forest floor litter and soil layers in natural forests and plantations at six sites across the eastern China. The error bars indicate standard deviation (n = 3). MH (Mohe), QY (Qingyuan), XY (Xinyang), HT (Huitong), DH (Dinghushan), JF (Jianfengling). *, ** and *** indicate a significant level at *p* < 0.05, *p* < 0.01 and *p* < 0.001, respectively.

Forest floor *δ*^13^C ranged between −31.58‰ and −27.73‰ in all sites sampled while forest floor *δ*^15^N ranged between −3.65‰ to 0.20‰ in NF, and between −4.66‰ to 3.66‰ in plantations. In general, forest floor *δ*^13^C values were significantly lower in NF than PF in XY and HT (Fig. 2) while the change in forest floor *δ*^15^N was not obvious except for DH and JF (Fig. 3). There were significant (*p* < 0.001) differences in forest floor *δ*^13^C and *δ*^15^N values among sites. In addition, LUC significantly increased forest floor *δ*^13^C values (Table 1) but not *δ*^15^N (*P* = 0.155).

Soil *δ*^13^C and *δ*^15^N values significantly (*P* < 0.001) varied among sites in mineral soil after the conversion from NF to PF. In addition, soil depth, LU and site × soil depth interaction were significantly altered after the conversion from NF to PF (Table 3). Soil *δ*^13^C and *δ*^15^N were enriched with depth after the conversion from NF to PF (Figs 2, 3), especially at the northern (MH and QY) and southern sites (DH and JF) indicating the high decomposition rate of soil organic matter (SOM). However, there were no differences in soil *δ*^13^C between NF and PF at QY and HT. Furthermore, soil *δ*^13^C was significantly different between NF and PF among 0–10 cm, 10–20 cm, 20–40 cm, 40–60 cm and 80–100 cm depths at DH and JF. Soil *δ*^15^N was enriched along soil profiles with the mean values of 2.98‰ in 0–10 cm, 4.29‰ in 10–20 cm and 5.35‰ in 20–40 cm but it decreased between 40–100 cm depth layers.

### Relationships of climatic factors to C and N contents, C/N ratios and δ^13^C and δ^15^N

**Figure 4:**
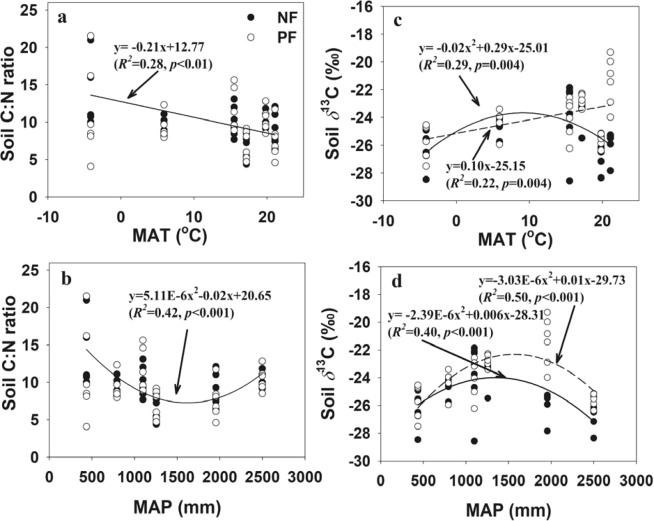
Regression relationships between (**a**) soil C/N ratios and mean annual temperature (MAT), (**b**) soil C/N ratios and mean annual precipitation (MAP), (**c**) soil *δ*^13^C and MAT, and (**d**) soil *δ*^13^C and MAP across forest type at 0–100 cm depth layer. The solid lines indicate regression relationships in natural forest stands, and the dotted lines indicate regression relationships in plantation stands. NF (Natural Forest), PF (Plantation Forest).

The mean annual temperature (MAT) ranged from −4.14 °C at MH to 21.08 °C at DH, and mean annual precipitation (MAP) increased from 436 mm to 2499 mm from northern to southern China (Table 5). We observed a linear relationship between soil C/N ratio and MAT (Fig. 4a), and a quadratic relationship between soil C/N ratio and MAP in NF (Fig. 4b). Soil *δ*^13^C were related to MAT and MAP in both NF and PF (Fig. 4c,d), with a linear relationship of soil *δ*^13^C and MAT in PF (*r* = 0.22, *P* = 0.004; Fig. 4c), and a quadratic relationship of soil *δ*^13^C and MAT in NF (*r* = 0.29, *P* = 0.004; Fig. 4c), and there were quadratic relationships of soil *δ*^13^C and MAP in both NF and PF (Fig. 4d).

**Table 5:** Location and characteristics of forest stands at six sites across the eastern China.

Sites	Location	pH		Elevation (m)	MAT (°C)	MAP (mm)	Climate zones	Natural land use	Plantation type	Plantation age
		NF	PF							
Mohe (MH)	N 52.92 E 122.79	5.1	5.1	448	−4.14	436	Cold temperate	CBM	*Larix gmelinii*	29
Qingyuan (QY)	N 41.85 E 124.93	5.5	5.5	597	5.91	794	Mid temperate	DBL	*Pinus koraiensis.*	38
Xinyang (XY)	N 31.77 E 114.03	5.7	5.1	189	15.49	1098	Northern subtropical	DEM	*Metasequoia glyptostroboides*	27
Huitong (HT)	N 26.85 E 109.60	4.58	4.6	427	17.17	1256	Mid subtropical	EBL	*Cunninghamia lanceolata*	33
Dinghushan (DH)	N 23.17 E 112.52	4.02	4.3	275	21.08	1955	Southern subtropical	MEB	*Pinus massoniana*	30
Jianfengling (JF)	N 18.44 E 108.01	4.52	4.6	800	19.80	2499	Tropical	TMF	*Pinus massoniana*	30

CBM (coniferous broad-leaf mixed forest), DBL (deciduous broad-leaved forest), DEM (deciduous evergreen mixed forest), EBL (evergreen broad-leaved forest), MEB (monsoon evergreen broad-leaved forest), TMF (tropical monsoon).

## Discussion

### Effects of forest conversion on carbon and nitrogen contents, and C/N ratios in forest floor and mineral soils

The present study provides a quantitative overview of C and N contents, and natural abundances of *δ*^13^C and *δ*^15^N in forest floor layer and mineral soil layers over 1-meter depth under NF and converted plantations across eastern China. Firstly, we found high forest floor N, but low C and C/N ratios in forest floor layer under NF as compared to PF. This point to the better quality of leaf litter materials in NF stands and large release potential of N during decomposition of the litter. This result was consistent with that found by Chen *et al*.^35^ who suggested the lower C/N ratios in forest floor litter in NF compared to hoop pine (*Araucaria cunninghamii*) plantations in Australia. The increase in forest floor C and C/N values and decrease in forest floor N may indicate that forest management practices provide favorable conditions for decomposing microorganisms thus alter their values as previously reported. In China, forest management practices have been directed towards timber production and sustained yield of wood supply, a practice which has significantly altered the balance between heterotrophic litter decomposition and litter inputs, thus impacting on the C content in the forest floor. This trend is consistent with the results of Vesterdal *et al*.^36^ who reported that the accumulation of nutrients in the forest floor is altered with increasing thinning intensity. There were significant differences in C, N and C/N within the study sites because of the effects of the forest conversion, including tree species that may affect the decomposition rate and the turnover of these elements in the soil^37,38^. Tree species differ in their carbon sequestration potential, LUC by changing species composition, tree density and forest structure altering their sequestration potential. Similar results have been reported by Vesterdal *et al*.^39^ who observed that forest floor C and N contents and C/N ratio were strongly affected by trees species. Besides, Fonseca *et al*.^37^ reported that forest floor C and N under the coniferous species had a large quantity of organic materials poorly decomposed, while a high rate of transformation of forest floor and incorporation in mineral soil have been observed in broad-leaved species. In our study, the forest floor litter was mainly composed of deciduous or evergreen broad-leaf litter in NF, while forest floor in PF is almost purely needle litter. Broad leaves are generally thought to produce mull forest floors that are richer in nutrients and promote rapid decomposition^40^. In addition, the higher forest floor N observed in NF at MH and QY is probably a result of the low temperature in North part of China which negatively affects the decomposition process and consequently leads a larger accumulation of organic matter. Thus, this study revealed that forest floor C and N content is strongly affected by human disturbances, consequently by LUC.

In mineral soil, C values were significantly altered after the conversion from NF to PF among site probably because of the difference of soil type, vegetation and trees species. The vegetation cover influences the storage of its elements in that it reduces the arrival of solar radiation directly to the soil. Yet, litter decomposition rates are controlled by the temperature and moisture which directly affect soil microbial activity. These findings are consistent with those of Jobbágy & Jackson^41^ who reported that the variation of soil C with depth in the profile varies strongly with vegetation type. Ramesh *et al*.^42^ also noted that quality and quantity of different soil organic carbon pools change with time depending on the rate of photosynthetic C addition and their losses through decomposition. Our study therefore suggested that LUC alters the carbon-holding capacity of soil in short carbon retention capacity of soil. Furthermore, the changes of soil C and N contents after NF converted to PF were not obvious. We observed a gain in soil C values at XY and HT sites and loss at DH, MH, JF, QY sites after the conversion from NF to PF suggesting that LUC from NF to PF influences C inputs. Similarly, Lewis *et al*.^43^ found that the effects of change from NF converted to introduced *Pinus* sp. plantations were highly site-specific and might have a positive, negative, or no influence on the variation of soil C values. Smith *et al*.^34^ also demonstrated that conversion from natural Amazonian forest to plantations altered soil organic C with an increase in surface under *Euxylophora paraensis* Hub. plantation and decreases under *Pinus caribaea* var*. honduensis* Barrett and Golfari. The result could be related to different quantity and quality of C input through root exudation, litter inputs and different management practices between NF and PF^44^. Although biological N fixation is the primary source of nitrogen input^45^, soil N values did not change significantly within the study sites after the conversion from NF to PF in the present study indicating that LUC did not alter significantly the balance between N input and loss.

In general, soil C and N where stored in the 0–20 cm depth segment of the overall profile, i.e. 67 and 57%, respectively. This is consistent with the results found by Batjes *et al*.^46^ who reported 50% the amount of OC located in the upper 30 cm of the soil organic carbon in the layer 0–100 cm. This result suggests a greater soil C sequestration within 0 to 20 cm soil depth. Furthermore, we found that soil C and N content decreased with soil depth may be due to the vertical distribution of roots. Indeed, Jobbágy *et al*.^41^, found that root distributions affect the vertical placement of C in the soil, and above- and below-ground allocation affects the relative amount of C that eventually falls to the soil surface from shoots. In addition, it well known that N status is a crucial factor driving forest soil C dynamics and high N availability can promote a greater soil C sequestration^13,47^. This trend is in line with our study which reported a significant and positive correlation between C and N probably through the effects on organic matter decomposition^45^.

### Effects of forest conversion on natural abundance of ^13^C and ^15^N in forest floor and mineral soils

Stable isotopic abundances of *δ*^13^C and *δ*^15^N have been used as powerful index to evaluate the long-term alterations of C and N cycles^16,17,19,20,22^. In the present study forest floor *δ*^13^C were enriched after LUC in subtropical and tropical regions while *δ*^15^N depleted. The depleted natural abundance of ^13^C and ^15^N in litterfall could account for low values of forest floor *δ*^13^C and *δ*^15^N in NF because of the generally higher above-ground litterfall input in NF compared to PF^7,48^. Moreover, we found that soil *δ*^13^C were strongly enriched at XY and HT sites after LUC. These findings might also be related to the less above-ground litterfall and below-ground roots inputs in these sites. Hertel *et al*.^49^ confirmed this trend by reporting that the conversion of tropical forest into plantations decrease C flux with fine root mortality to soil organic C pool. Moreover, soil organic C and N accumulation and stability have been suggested to be strongly influenced by litter quality, resulting in more stable organic C accumulated in NF soils with high quality of litter substrates^50^. On the other hand, the intensive management practices in PF (e.g., clear-cutting and slash burning, site preparation and pruning) accompanied by higher temperature and precipitation conditions in tropical and subtropical (at the DH and JF sites) regions could enrich soil ^13^C and ^15^N in plantation because of more soil C and N loss^27,44^. However, soil organic C accumulation and stability were also influenced by soil matrix structure^51^, and were related to the saturation of SOC, climatic zones and ecosystem types^52^. It was difficult to explain clearly the effect of LUC on soil *δ*^15^N variation within the study sites because of the complex interactions between abiotic and biotic factors.

A number of studies observed an increasing tendency of *δ*^13^C and *δ*^15^N with soil depth^20,21,23,53^. In our study, we observed an enrichment of soil *δ*^13^C following the increase of depth layer by 3.09‰ in surface mineral soil (0–10 cm) after LUC probably because deeper soil layer has a greater humification of organic matter decomposition or largely to the increasing residence time of the organic C in the soil^54^. This result was in line with Brunn *et al*.^20^ who found the mean ^13^C enrichment between *δ*^13^C values of the Oi horizon and *δ*^13^C values of soil organic matter (SOM) in 10 cm soil depth was 3.4 + 0.2‰ under temperate beech forests. Several processes might cause enrichment of *δ*^13^C with increasing soil depth e.g., the depletion of ^13^C in the atmosphere due to combustion of fossil fuel, and the considerable presence of litter and roots with depleted ^13^C in the upper soil. Accoe *et al*.^55^ observed that the average rate of change in soil *δ*^13^C is directly related to organic matter decomposition rates in different parts of the soil profile. Thus, the extent of change in ^13^C-abundance with increasing soil depth may indicate the quality or stability of SOM under continuous C3 vegetation^55^. Furthermore, isotopic fractionation during microbial metabolism of SOM and the downward cycling of hydrophilic ^13^C enriched decomposing products with dissolved organic carbon fluxes possibly contributing to the establishment of the vertical *δ*^13^C depth trends^54,56^. The pattern of soil *δ*^13^C observed in PF of DH showed an increase from 0 to 40 cm and then a decrease until 100 cm depth layer reflecting the more complex processes of microbial degradation and mixing of soil C of different ages^57^. On the other hand, the low soil *δ*^15^N values observed at the surface soil across the study sites particularly at XY could be due to the continuous addition of plant residues with extremely low *δ*^15^N values^58^.

### Effects of climatic factors on carbon, nitrogen and their isotopic abundances

Climatic variables have strong impacts on soil organic C and N contents^59,60^, and natural abundance of soil ^13^C^20,22^ and ^15^N^61,62^. There were linear and non-linear regressions of soil C/N ratios and *δ*^13^C with MAT and MAP in both NF and plantations, which indicated that SOC turnover rates were largely determined by MAT and MAP. Our result was consistent with Wang *et al*.^22^ who examined large-scale controls of climate over patterns of SOC turnover across terrestrial biomes worldwide using a meta-analysis of soil *δ*^13^C in previous literatures and demonstrated that SOC turnover was substantially accelerated with increasing MAT. In addition, the distribution of soil *δ*^13^C was slightly correlated with MAP after LUC probably due to the replacement of vegetation types which determines changes in the relative vertical distribution of soil C along rainfall gradients^41^ after LUC. This result is in line with those found by Burke *et al*.^63^ who reported that precipitation clearly has a direct role regionally and globally in the amount of soil C stored. Thus, our findings revealed that MAP is a key factor controlling soil C accumulation and decomposition after the conversion from NF to PF. According to Jia *et al*.^64^, vegetation type and soil type rather than MAT explained the variability in soil *δ*^13^C along the 400 mm isohyet in China.

Previous studies suggested that climate is the primary factor controlling soil *δ*^15^N^61^. Contrary to these results, we did not observe clear relationship between climate factors and *δ*^15^N in the present study. This could be due to the climate change which may change soil *δ*^15^N values through altered precipitation patterns, elevated temperatures, and more frequent and extreme weather events. In addition, climate change can affect temperature-sensitive biogeochemical processes, including N mineralization, nitrification and denitrification, soil respiration, litter decomposition, as well as root dynamics and plant productivity^65^, consequently, altering the rate of *δ*^15^N accumulation.

## Materials and Methods

### Study sites and soil sampling

**Figure 5:**
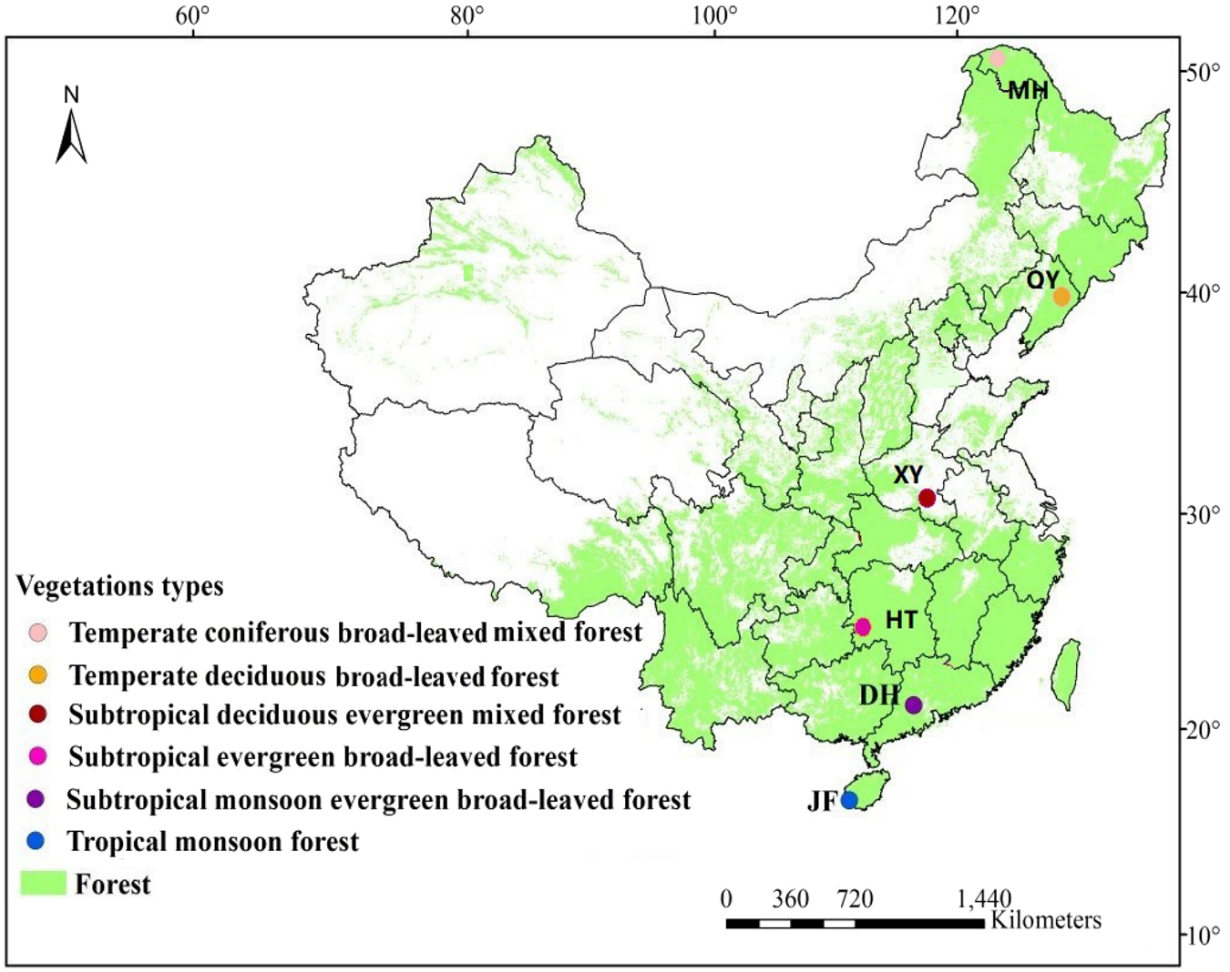
The location of forest stands at six sites across eastern China. MH (Mohe), QY (Qingyuan), XY (Xinyang), HT (Huitong), DH (Dinghushan), JF (Jianfengling).

In August 2017, soil samples were collected from one natural forest (NF) and one plantation forest (PF) stand at six sites including Mohe (MH), Qingyuan (QY), Xinyang (XY), Huitong (HT), Dinghushan (DH), Jianfengling (JF) across eastern China forest (Fig. 5, Table 5). To minimize the impact of local terrain on vegetation or trees, the topography of all selected plots was uniform and flat. In each one of these, the forest floor litter was collected using three 10 cm × 10 cm wooden frames in each stand. Sampling was done carefully in order to avoid contamination with the mineral material. Forest floors were very thin in most stands and were dried at 65 °C for 72 h and roots were sorted out and weighed to determine dry mass. Mineral soils were sampled in the same points where forest floors had been removed. For soil sampling, three pits were dug at 1-meter depth, and mineral soil samples were collected at 0–10, 10–20, 20–40, 40–60, 60–80 and 80–100 cm along soil profiles. A total of 252 samples were collected from 6 sites × 2 land uses × 3 pits × 7 layers. Mean annual temperature (MAT) and mean annual precipitation (MAP) data at six sites were recorded from the adjacent climate observing stations during 1960–2014.

### Soil C and N contents, and δ^13^C and δ^15^N analysis

Forest floor litter samples were oven-dried at 65 °C for 72 h, and mineral soil samples were air-dried at room temperature for 2 weeks before passing through a 2 mm sieve to remove coarse stones, plant residues, and roots. Then, the forest floor and soil samples were ground to fine powders using a ball mill (JXFSTPRP-64, Jingxin Co., Ltd, China) for measurements of soil C and N contents, and *δ*^13^C and *δ*^15^N.

Soil C and N pools and stable isotope ^13^C and ^15^N composition were measured using an isotope ratio mass spectrometer (IRMS) (IsoPrime 100, Isoprime Ltd., UK), connected to a CN elemental analyzer (Vario MICRO cube, Elementar, Germany). Carbon and nitrogen stable isotope abundances were calculated as *δ*^13^C and *δ*^15^N (‰) using the following formula:

$${\delta }^{13}{\rm{C}}\,{\rm{or}}\,{\delta }^{15}{\rm{N}}(\textperthousand )=({{\rm{R}}}_{{\rm{sample}}}/{{\rm{R}}}_{{\rm{standard}}}\,-\,1)\,\ast \,1000,$$

where R_sample_ is the^13^C:^12^C or ^15^N:^14^N ratio in the samples and R_standard_ is the ^13^C:^12^C or ^15^N:^14^N ratio in the standard. The Vienna Pee Dee Belemnite (VPDB) and atmospheric N_2_ (*δ*^15^N = 0.0‰) were used as the standard, respectively. The precision of isotopic composition was checked using internal standards i.e. acetanilide, L-histidine, D-glutamic and glycine^28^. In general, the analytical precision for *δ*^13^C and *δ*^15^N was better than 0.2‰.

### Statistical analysis

The results are the average of the replicates determined from three subsamples of the same site. Statistically significant differences were determined by *P* < 0.05 unless otherwise stated. The sample differences of *δ*^15^N, *δ*^13^C values, C and N concentrations, the effect of LU and depth on C, N, *δ*^13^C, and *δ*^15^N in whole soil and the comparison of C and N among LU types and depths were tested with analysis of multivariance (ANOVA). Linear regression and nonlinear regression analysis were used to test the relationships of C/N, soil *δ*^13^C with climatic factors (MAT, MAP) across forest type. The samples from each depth were used as dependent measurements in the regression. All statistical analyses were performed with the SPSS version 20.0 (Systat Statistical Software Package for Windows)^66^.
